# Interictal intracranial EEG asymmetry lateralizes temporal lobe epilepsy

**DOI:** 10.1093/braincomms/fcae284

**Published:** 2024-08-22

**Authors:** Erin C Conrad, Alfredo Lucas, William K S Ojemann, Carlos A Aguila, Marissa Mojena, Joshua J LaRocque, Akash R Pattnaik, Ryan Gallagher, Adam Greenblatt, Ashley Tranquille, Alexandra Parashos, Ezequiel Gleichgerrcht, Leonardo Bonilha, Brian Litt, Saurabh R Sinha, Lyle Ungar, Kathryn A Davis

**Affiliations:** Department of Neurology, Perelman School of Medicine, University of Pennsylvania, Philadelphia, PA 19104, USA; Center for Neuroengineering and Therapeutics, University of Pennsylvania, Philadelphia, PA 19104, USA; Center for Neuroengineering and Therapeutics, University of Pennsylvania, Philadelphia, PA 19104, USA; Department of Bioengineering, School of Engineering & Applied Sciences, University of Pennsylvania, Philadelphia, PA 19104, USA; Perelman School of Medicine, University of Pennsylvania, Philadelphia, PA 19104, USA; Center for Neuroengineering and Therapeutics, University of Pennsylvania, Philadelphia, PA 19104, USA; Department of Bioengineering, School of Engineering & Applied Sciences, University of Pennsylvania, Philadelphia, PA 19104, USA; Center for Neuroengineering and Therapeutics, University of Pennsylvania, Philadelphia, PA 19104, USA; Department of Bioengineering, School of Engineering & Applied Sciences, University of Pennsylvania, Philadelphia, PA 19104, USA; Center for Neuroengineering and Therapeutics, University of Pennsylvania, Philadelphia, PA 19104, USA; Department of Neurology, Perelman School of Medicine, University of Pennsylvania, Philadelphia, PA 19104, USA; Center for Neuroengineering and Therapeutics, University of Pennsylvania, Philadelphia, PA 19104, USA; Center for Neuroengineering and Therapeutics, University of Pennsylvania, Philadelphia, PA 19104, USA; Department of Bioengineering, School of Engineering & Applied Sciences, University of Pennsylvania, Philadelphia, PA 19104, USA; Center for Neuroengineering and Therapeutics, University of Pennsylvania, Philadelphia, PA 19104, USA; Perelman School of Medicine, University of Pennsylvania, Philadelphia, PA 19104, USA; Department of Neurology, Washington University in St. Louis, St. Louis, MO 63110, USA; Center for Neuroengineering and Therapeutics, University of Pennsylvania, Philadelphia, PA 19104, USA; Department of Neurology, Medical University of South Carolina, Charleston, SC 29425, USA; Department of Neurology, Emory University, Atlanta, GA 30325, USA; Department of Neurology, Emory University, Atlanta, GA 30325, USA; Department of Neurology, Perelman School of Medicine, University of Pennsylvania, Philadelphia, PA 19104, USA; Center for Neuroengineering and Therapeutics, University of Pennsylvania, Philadelphia, PA 19104, USA; Department of Bioengineering, School of Engineering & Applied Sciences, University of Pennsylvania, Philadelphia, PA 19104, USA; Department of Neurology, Perelman School of Medicine, University of Pennsylvania, Philadelphia, PA 19104, USA; Department of Computer and Information Science, University of Pennsylvania, Philadelphia, PA 19104, USA; Department of Neurology, Perelman School of Medicine, University of Pennsylvania, Philadelphia, PA 19104, USA; Center for Neuroengineering and Therapeutics, University of Pennsylvania, Philadelphia, PA 19104, USA

**Keywords:** EEG, intracranial EEG, temporal lobe epilepsy, interictal EEG

## Abstract

Patients with drug-resistant temporal lobe epilepsy often undergo intracranial EEG recording to capture multiple seizures in order to lateralize the seizure onset zone. This process is associated with morbidity and often ends in postoperative seizure recurrence. Abundant interictal (between-seizure) data are captured during this process, but these data currently play a small role in surgical planning. Our objective was to predict the laterality of the seizure onset zone using interictal intracranial EEG data in patients with temporal lobe epilepsy. We performed a retrospective cohort study (single-centre study for model development; two-centre study for model validation). We studied patients with temporal lobe epilepsy undergoing intracranial EEG at the University of Pennsylvania (internal cohort) and the Medical University of South Carolina (external cohort) between 2015 and 2022. We developed a logistic regression model to predict seizure onset zone laterality using several interictal EEG features derived from recent publications. We compared the concordance between the model-predicted seizure onset zone laterality and the side of surgery between patients with good and poor surgical outcomes. Forty-seven patients (30 female; ages 20–69; 20 left-sided, 10 right-sided and 17 bilateral seizure onsets) were analysed for model development and internal validation. Nineteen patients (10 female; ages 23–73; 5 left-sided, 10 right-sided, 4 bilateral) were analysed for external validation. The internal cohort cross-validated area under the curve for a model trained using spike rates was 0.83 for a model predicting left-sided seizure onset and 0.68 for a model predicting right-sided seizure onset. Balanced accuracies in the external cohort were 79.3% and 78.9% for the left- and right-sided predictions, respectively. The predicted concordance between the laterality of the seizure onset zone and the side of surgery was higher in patients with good surgical outcome. We replicated the finding that right temporal lobe epilepsy was harder to distinguish in a separate modality of resting-state functional MRI. In conclusion, interictal EEG signatures are distinct across seizure onset zone lateralities. Left-sided seizure onsets are easier to distinguish than right-sided onsets. A model trained on spike rates accurately identifies patients with left-sided seizure onset zones and predicts surgical outcome. A potential clinical application of these findings could be to either support or oppose a hypothesis of unilateral temporal lobe epilepsy when deciding to pursue surgical resection or ablation as opposed to device implantation.

## Teaser

Conrad *et al*. report that quantitative analysis of interictal intracranial EEG data predicts the laterality of the seizure onset zone in patients with drug-resistant temporal lobe epilepsy. Left-sided seizure onsets are easier to distinguish, and a simple model using just spike rates outperforms a more complex model.

## Graphical Abstract

**Graphical Abstract:**
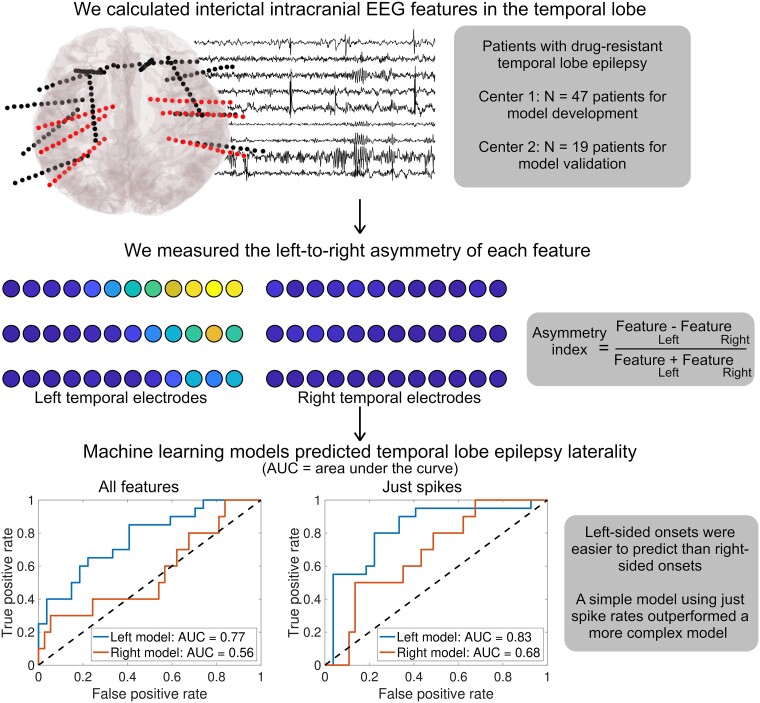

## Introduction

Temporal lobe epilepsy (TLE) is the most common localization of drug-resistant epilepsy in adults.^1^ Determining the laterality of TLE—left, right, or bilateral—is a common clinical question that dictates the type and location of surgery. For patients with suspected TLE in which the laterality or localization is uncertain, one standard clinical approach is to implant intracranial electrodes and wait 1–2 weeks for the patient to have multiple seizures.^2,3^ This process is time-consuming and exposes the patient to morbidity associated with electrode implantation, recurrent seizures and prolonged hospitalization. Ironically, it may also be too short to determine epilepsy laterality: data from chronic intracranial EEG recordings reveal that, in patients with bilateral seizure onsets, it often takes several weeks to months to capture the first contralateral seizure.^4^ Even in the setting of weaning medications in the Epilepsy Monitoring Unit, patients with a moderate pre-test probability of multifocal seizures may need seven or more seizures to achieve a high posttest probability of unifocal epilepsy, which is more seizures than can often be obtained in a 1–2 week intracranial EEG evaluation.^5^

Acknowledging the limitations of ictal EEG data, clinicians also use multiple sources of ancillary data in surgical planning. These data include seizure semiology, imaging such as MRI and PET, as well as ‘interictal EEG data’, from the time period in between the seizures.^6^ The primary feature of the interictal EEG used in surgical planning is interictal spikes. There is evidence that resecting regions with frequent spikes is associated with good surgical outcomes.^7-10^ Additional interictal features, such as bandpower and connectivity, have been proposed as potential biomarkers of seizure generators.^11-17^ A major challenge that currently limits the use of interictal intracranial EEG data in surgical planning is that, up until recently, we have lacked methods to interpret such large quantities of data.

In this study, we hypothesized that features of the interictal EEG differ between patients with left-sided, right-sided and bilateral TLE. We studied interictal intracranial EEG data from patients with drug-resistant TLE. We calculated several interictal EEG features that have been reported to localize the seizure onset zone (SOZ) in prior studies, and compared these features between patients with left, right and bilateral TLE. Next, in order to understand if lateralizing interictal features exist across modalities, we studied resting-state functional MRI (fMRI) connectivity in a separate group of patients with TLE. Finally, we developed a machine learning algorithm to predict the clinician-defined seizure onset zone laterality using interictal data, and then validated this model across patients from two epilepsy centres.

## Materials and methods

### Patient selection

This retrospective study was approved by the Institutional Review Boards of the Hospital of the University of Pennsylvania (HUP) and Medical University of South Carolina (MUSC). We analysed sequential patients with drug-resistant epilepsy who underwent intracranial EEG recording as part of surgical evaluation (HUP date range: 2013–2021; MUSC date range 2021–2022), consented to analysis of their intracranial data and who met the following inclusion criteria: (i) clinical determination of TLE; and (ii) bilateral electrode coverage of the temporal lobes. Details on electrode configurations, recording, and determination of seizure onset zone and surgical outcome are in the Supplementary Methods. Seizure times and the anatomical seizure onset localization were identified by a board-certified epileptologist reviewing the EEG record for clinical purposes, and confirmed in a clinical case conference consisting of multiple epileptologists. The determination of the seizure onset localization was a consensus clinical designation, which at our centre is typically made by confirming consistent localization and laterality of seizure onset across multiple clinically habitual seizures. A board-certified neurologist (E.C.C. at HUP; A.P. at MUSC) also reviewed the surgical conference notes and designated whether the patients’ MRIs were determined to have a lesion in the temporal lobes. For patients who underwent resection or ablation, we measured surgical outcome at one year post-surgery using both the ILAE and Engel classification scales.^18,19^ Table 1 shows clinical information for all patients.

**Table 1:** Clinical information

	HUP IEEG	MUSC IEEG	HUP fMRI
Total: *n*	47	19	62
Female: *n* (%)	30 (63.8%)	10 (52.6%)	33 (53.2%)
Age at onset in years: median (range)	16.0 (0.9–59.0)	25.0 (8.0–59.0)	20.0 (0.3–61.0)
Age at implant in years: median (range)	38.0 (20.0–69.0)	36.5 (23.0–73.0)	35.3 (18.9–70.4)
MRI with temporal lesion: *n* (%)	14 (29.8%)	13 (68.4%)	37 (59.7%)
Bilateral or discordant pre-implant hypotheses: *n* (%)	33 (70.2%)	17 (89.5%)	NA
Symmetric mesial temporal-targeted contacts: median (range)	44.0 (8.0–72.0)	60.0 (20.0–60.0)	NA
SOZ lateralization (clinician determination)			
Left: *n* (%)	20 (42.6%)	5 (26.3%)	30 (48.4%)
Right: *n* (%)	10 (21.3%)	10 (52.6%)	17 (27.4%)
Bilateral: *n* (%)	17 (36.2%)	4 (21.1%)	15 (24.2%)
SOZ localization (clinician determination)			
Mesial temporal: *n* (%)	27 (57.4%)	15 (78.9%)	
Temporal neocortical: *n* (%)	6 (12.8%)	3 (15.8%)	
Broad temporal: *n* (%)	14 (29.8%)	1 (5.3%)	
Surgery performed			
Resection: *n* (%)	8 (17.0%)	5 (26.3%)	10 (16.1%)
Ablation: *n* (%)	17 (36.2%)	2 (10.5%)	16 (25.8%)
Device: *n* (%)	14 (29.8%)	6 (31.6%)	9 (14.5%)
Engel 1 year outcome	*n* = 24 with outcomes	*n* = 2 with outcomes	*n* = 13 with outcomes
Median (range)	1.0 (1.0–4.0)	1.0 (1.0–1.0)	1.0 (1.0–3.0)
ILAE 1 year outcome	*n* = 24 with outcomes	*n* = 2 with outcomes	*n* = 13 with outcomes
Median (range)	2.0 (1.0–5.0)	1.0 (1.0–1.0)	3.0 (1.0–5.0)

The first column shows the clinical variables, the second column shows the data for the cohort undergoing intracranial EEG (IEEG) at HUP, the third column shows data for the cohort undergoing IEEG at MUSC used for external model validation, and the fourth column shows data for the cohort undergoing fMRI at HUP. The ‘Bilateral or discordant pre-implant hypotheses’ variable indicates the number of patients for whom one of the leading two pre-implant localization hypotheses was bilateral, or for whom the leading two pre-implant hypotheses had discordant lateralities (e.g. the top hypothesis was left temporal lobe epilepsy and the second hypothesis was right temporal lobe epilepsy). Patients included in the outcome analysis are those who underwent resection or ablation and had at least one year of follow-up.

### Intracranial EEG pre-processing, feature selection and asymmetry index calculation

**Figure 1:**
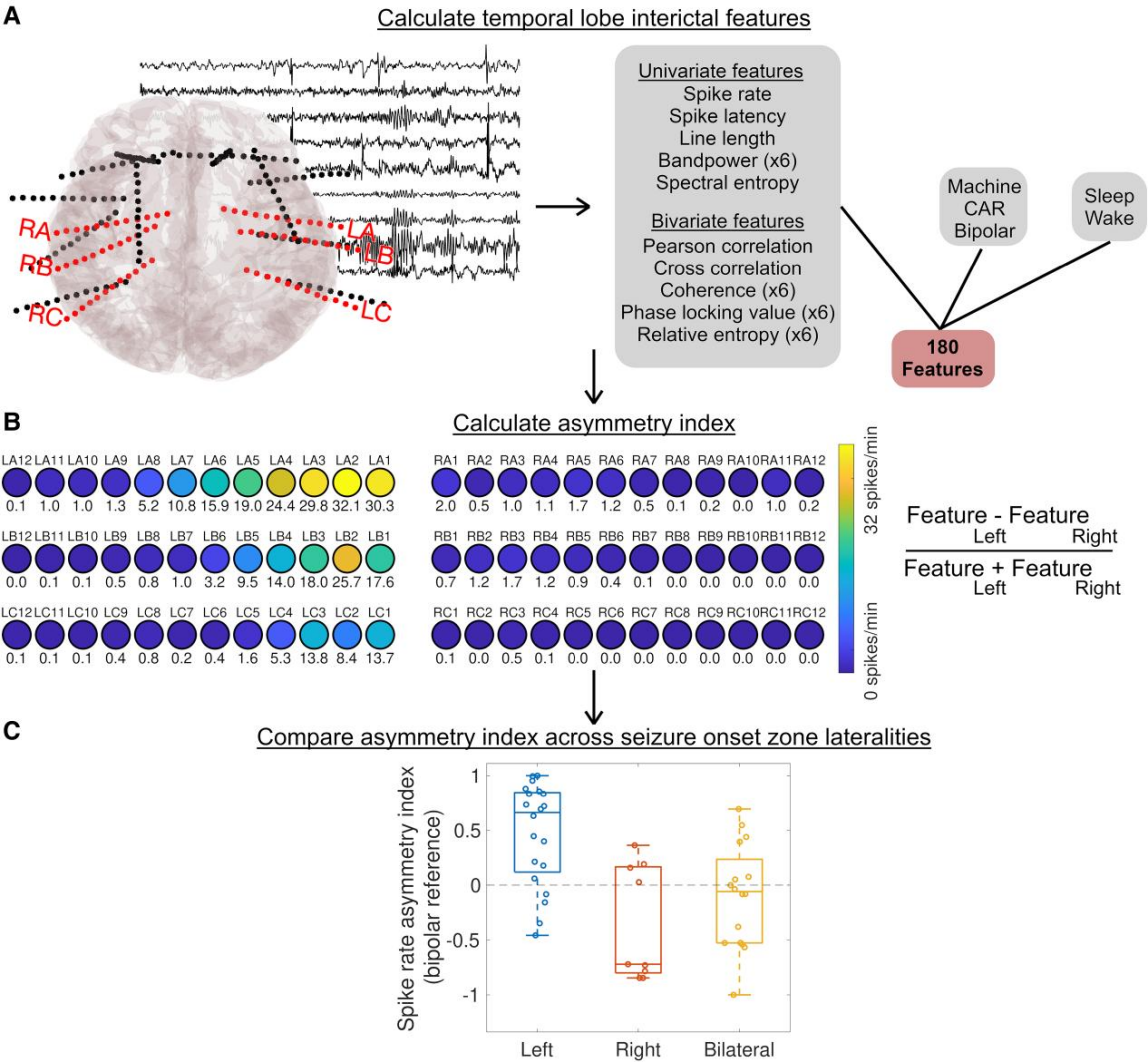
**Methods to measure interictal IEEG asymmetry index (AI).** (**A**) We identified electrodes targeting the bilateral temporal structures, and calculated several univariate and bivariate (connectivity) features for three choices of reference [machine, common average (CAR) and bipolar] and in both sleep and wake, resulting in a total of 180 features. (**B**) We calculated the asymmetry index for each feature. In this example, interictal spike rates (spikes/minute) detected in a common average montage during sleep are shown in the temporal electrodes for a single patient. Brighter colours indicate higher spike rates. LA, LB, LC, RA, RB and RC are the names of six different electrodes (with 12 contacts each) targeting temporal lobe structures (lower numbered contacts are more mesial). Spike rates in spikes/minute are denoted below the corresponding electrode contacts. (**C**) We compared the asymmetry index across patients with different seizure onset zone lateralities. Each circle represents a separate patient at HUP. Blue dots (first column) = left TLE; red (middle column) = right TLE; yellow (third column) = bilateral TLE. In this case, asymmetry indices for spike rates in sleep, detected in a bipolar reference, are shown.

A full description of temporal and spatial sampling is in the Supplemental Methods. Briefly, we selected a continuous 12-hour period of EEG data and applied the SleepSEEG algorithm^20^ to determine sleep stage. We next divided the 12-hour period of EEG into 72 10-minute segments, and then selected a random 1-minute segment from each 10-minute segment (the random selection was performed by selecting a uniformly-distributed pseudorandom integer as the starting time sample, using Matlab's randi function). We excluded time periods overlapping with any seizures noted in the clinical record. We removed non-temporal lobe electrode contacts and those contacts lacking a contralateral pair (Fig. 1A) because analysing symmetric electrode pairs limits the confounder of inter-contact distance affecting functional connectivity measurements.^21,22^ We identified and eliminated channels with artifacts^23^ and employed three different referencing methods: machine reference, common average reference and bipolar reference (referencing was performed after removing non-symmetric and non-temporal lobe contacts). We applied a 60 Hz notch filter and a 0.5–80 Hz bandpass filter, chosen to agree with methods from related publications of intracranial EEG.^24,25^

We calculated several features, chosen based on their prior use in the literature for identifying the seizure onset zone (Fig. 1A and Table 2). The Supplementary Materials fully describe the calculation of each feature. Briefly, these features included spike rates and spike timing detected using a previously validated automated detector^26^ univariate features such as bandpower,^27^ line length^28^ and spectral entropy;^29^ and several bivariate features representing functional connectivity, including Pearson correlation,^22^ coherence,^30^ cross-correlation^31^ and phase-locking value,^32^ and relative entropy.^13,16^ Some features were calculated only over the broadband signal, and some were also calculated over the EEG signal filtered to five canonical frequencies, following choices previously published in the literature. We separately examined features in N2/N3 sleep and wake (these stages were found to have excellent performance in the original SleepSEEG validation study).

**Table 2:** EEG features

Feature	Univariate or bivariate	Frequency	Conceptual definition
Line length	Univariate	Broadband	The sum of the absolute difference between the EEG signal at adjacent time points
Bandpower	Univariate	Broadband + canonical	The EEG signal power in a given frequency band
Spike rate	Univariate	Broadband	The frequency of interictal spikes
Spike recruitment latency	Univariate	Broadband	The latency of an interictal spike in a sequence (where the first spike in a sequence has a latency of 0 ms)
Spectral entropy	Univariate	Broadband	The level of uncertainty in an EEG spectral power distribution (how similar the signal is to white noise)
Pearson correlation	Bivariate	Broadband	The squared linear correlation between the EEG signals on a pair of electrode contacts
Cross correlation	Bivariate	Broadband	The maximum absolute value linear correlation between the time-shifted EEG signals on a pair of electrode contacts
Coherence	Bivariate	Broadband + canonical	The frequency-specific correlation between the EEG signals on a pair of electrode contacts
Phase-locking value (PLV)	Bivariate	Broadband + canonical	The phase synchrony between the EEG signals on a pair of electrode contacts
Relative entropy	Bivariate	Broadband + canonical	The difference between the probability distributions of the EEG signals on a pair of electrode contacts

All features studied, their classification as univariate (describing the signal on a single electrode contact) or bivariate (describing the relationship between the signals on a pair of electrode contacts), the frequency ranges over which they were calculated, and their conceptual definitions are shown. Each of the 10 features was calculated across the corresponding number of frequencies shown in the table, across three choices of reference and across wake and sleep, yielding a total of (6 single frequency features + 4 multi-frequency features × 6 frequency bands) × 3 × 2 = 180 features.

We next calculated the ‘asymmetry index’ (AI) for each feature, defined as:


$$\mathrm{AI}=\frac{\mathrm{Featur}e_{\mathrm{Left}}-\;\mathrm{Featur}e_{\mathrm{Right}}}{\mathrm{Featur}e_{\mathrm{Left}}+\mathrm{Featur}e_{\mathrm{Right}}}$$

where Feature_Left_ is the mean feature across left temporal lobe contacts, and Feature_Right_ is the mean feature across right temporal lobe contacts. A positive AI implies higher values of the feature on the left, and a negative AI implies higher values of the feature on the right.

### fMRI analysis

To determine if interictal asymmetries from non-EEG modalities also lateralize TLE, we studied resting-state fMRI connectivity using the dataset and processing techniques from a recent paper published by our group.^33^ Subjects were 62 patients at HUP with drug-resistant TLE, six of whom overlapped with those in our intracranial EEG cohort. We calculated the average BOLD time series for each voxel within each parcel of the Brainnetome atlas^34^ and created a functional connectivity matrix by computing the absolute value of the Pearson correlation between the time series in each pair of parcels. We identified Brainnetome parcels belonging to temporal lobe grey matter structures (Supplementary Table 1), and measured the average left and right temporal connectivity. We defined AI as:


$$\mathrm{AI}\;=\;\frac{\mathrm{Connectivit}y_{\mathrm{Left}}\;-\;\mathrm{Connectivit}y_{\mathrm{Right}}}{\mathrm{Connectivit}y_{\mathrm{Left}}\;+\;\mathrm{Connectivit}y_{\mathrm{Right}}}$$

where Connectivity_Left_ is the mean connectivity in the left temporal lobe, and Connectivity_Right_ is the mean connectivity in the right temporal lobe. Based on our prior findings that the epileptic hemisphere demonstrates broad holo-hemispheric reductions in intracranial EEG connectivity, we hypothesized that resting-state fMRI connectivity would also be lower in the epileptic temporal lobe.^21^

### Machine learning to predict SOZ laterality

Detailed steps are in the Supplemental Methods. Briefly, to separately examine the ease of predicting left versus right SOZ laterality, we built two classifiers: one predicting left-sided SOZ (as opposed to either right ‘or’ bilateral), and one predicting right-sided SOZ (as opposed to left or bilateral), both using interictal EEG AI values as features. Missing features were estimated by median imputation. For each classifier, we performed principal component analysis (PCA) on the training data, retaining enough components to explain 95% of the variance in the features. We performed LASSO logistic regression (with *λ* equal to 1/*N*, where *N* is the training sample size) on the preserved principal components. For each classifier, we performed internal validation on the HUP dataset using leave-one-patient-out cross-validation, and then external validation on the MUSC dataset, trained on the full HUP dataset. To estimate feature importance, we multiplied the PCA transformation matrix by the classifier feature weights in order to derive standardized weights for the original set of AI features.^32^

We next developed two single-feature models: the first included only mean spike rate AI. This model was developed to test whether spike rate asymmetry alone could successfully lateralize TLE. The second single-feature model included ‘binarized’ mean spike rate AI, with the input being 1 if the spike rate AI was positive and 0 if negative. This model approximated a qualitative clinical approach of considering which side has more spikes, rather than the quantitative difference between sides. We primarily analysed spikes detected in common average reference, with secondary analyses of bipolar and machine references to test the sensitivity of the results to this choice. In order to have a single feature, we also chose to only study spikes in sleep given work suggesting that interictal features in sleep better localize seizure generators than features in wakefulness.^13,35^ We next examined whether model accuracy was associated with the precise localization of the SOZ. We aggregated patients from both HUP and MUSC, and selected only those patients whose clinician-defined SOZ was either mesial temporal (*n* = 42) or temporal neocortical (*n* = 9), excluding 15 patients with broader temporal localizations. We tested for the association between mesial temporal versus temporal neocortical localization and correct versus incorrect laterality classification with a Fisher's exact test. We also used a Fisher's exact test to test for the association between model accuracy and whether the patient had a temporal lobe lesion on MRI (*n* = 27) as opposed to other lesions or no lesion (*n* = 39).

Clinician determinations of SOZ laterality may be inaccurate. Given our hypothesis that interictal features reveal seizure generators, we predicted that our models would perform better in those with good surgical outcomes. To test this, we examined the subset of all patients from our larger cohort who underwent either temporal lobe resection or ablation and had at least one year of surgical follow-up. For each patient, we identified the side of surgery, and we measured the spike rate model-predicted probability of SOZ laterality on that side. We selected the model (left versus right/bilateral, or right versus left/bilateral) corresponding to the side of surgery. We compared model probabilities between patients with a good surgical outcome and those with a poor surgical outcome. Finally, we constructed a logistic regression model using the model-predicted probability of SOZ laterality on the side of surgery as the sole predictor, and the outcome (good or poor) as the binary response variable. We validated this classifier using leave-one-out cross-validation.

### Statistical analysis

For univariate analyses, we report mean and standard deviation (SD). To compare two paired or independent groups, we report *t*-tests (alpha = 0.05) and Cohen *d* for effect size. To compare more than two groups, we report ANOVA tests and *η*^2^ for effect size. The Benjamini–Hochberg procedure was applied to control the false discovery rate at alpha = 0.05.^36^ Analyses were performed in Matlab R2022a (Mathworks).

## Results

We examined 47 patients at HUP for model development and internal validation, and 19 patients at MUSC for external model validation (Table 1). TLE lateralities were imbalanced across the two centres, with left TLE being more prevalent at HUP (42.6% left, 21.3% right, 36.2% bilateral), and right TLE being more prevalent at MUSC (26.3% left, 52.6% right, 21.1% bilateral). We visually validated a random sample of 50 automated spike detections from each patient (bipolar montage). The median [interquartile range (IQR)] percentage of automatically-detected spikes determined to be true spikes was 90.0% (82.0–95.5%) for HUP and 90.0% (76.0–94.0%) for MUSC. Supplementary Fig. 1 shows 25 random spike detections from two patients from HUP and two patients from MUSC, chosen as representative examples because the positive predictive values of their spike detections were at the bottom and top of the interquartile range across all patients from each centre. Of the 72 time segments studied per patient, a median of 19.5 (IQR 12.0–23.0) were determined to represent wakefulness, and 28.0 (IQR 22.0–31.0) were determined to be in N2 or N3 sleep (remaining segments were determined to be in other sleep stages, or occurring during a transition between sleep stages).

### Asymmetries in spike rates and relative entropy are distinct across SOZ lateralities

**Figure 2:**
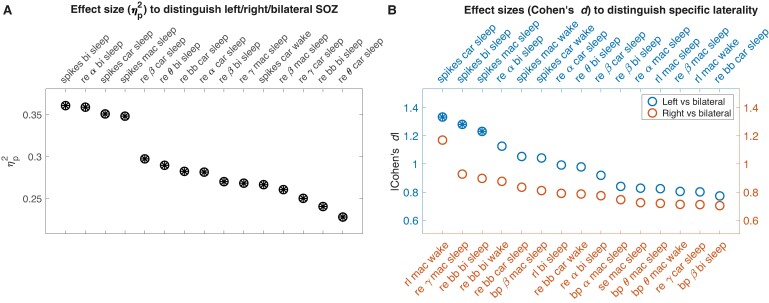
**Comparison of IEEG asymmetry index (AI) across seizure onset zone (SOZ) lateralities.** (**A**) Effect sizes (*η*^2^) at distinguishing patients with different SOZ lateralities for the 15 features with the highest effect sizes. Circles filled with asterisks (all in this plot) represent features that significantly distinguished SOZ lateralities (ANOVA with Benjamini–Hochberg false discovery rate correction). (**B**) Effect sizes (absolute value of Cohen's *d*) at distinguishing SOZ lateralities for the 15 features with the highest effect sizes at distinguishing left from bilateral SOZs and right from bilateral SOZs, respectively. Note that in this analysis, as opposed to that for Fig. 4, we separated left from bilateral and right from bilateral, rather than left from right/bilateral and right from left/bilateral. We did this to achieve distinct groups to better understand the separate univariate features that distinguished left and right TLE. IEEG AI distinguished left from bilateral SOZs more easily than right from bilateral SOZs. bi, bipolar reference; car, common average reference; mac, machine reference; spikes, spike rates; re, relative entropy; rl, spike recruitment latency (a measure of spike timing); bp, bandpower; se, spectral entropy. Greek letters indicate canonical frequency bands. bb, broadband. IEEG analyses were performed in the HUP cohort to preserve the MUSC cohort data for external model validation. Patient numbers vary slightly across analyses and are listed along with corresponding statistics in Supplementary Tables 2–4.

We compared interictal EEG feature AIs between patients with left-sided, right-sided and bilateral SOZs. We ranked features in descending order by effect size (*η*^2^) at separating the three SOZ lateralities (Fig. 2A). The top-ranked AI features involved spike rates and relative entropy, and were predominantly in sleep. For example, spike rates in sleep (bipolar reference) had the highest effect size (ANOVA: *F*(2,43) = 12.1, *P* < 0.001, *q* = 0.004, *η*^2^ = 0.36). Supplementary Table 2 shows the corresponding statistics for the features with the 30 highest effect sizes. We next compared the set of features that best distinguished left from bilateral SOZs versus right from bilateral SOZs (Fig. 2B). Spike features significantly distinguished left from bilateral SOZs. For distinguishing right-sided SOZ, several other features performed best (though none were significant after correcting for the false discovery rate). Supplementary Tables 3 and 4 show the effect sizes and significance levels for the features with the highest effect sizes at differentiating left from bilateral SOZs and right from bilateral SOZs, respectively. This suggests that right-sided SOZs are harder to distinguish than left-sided SOZs in our dataset. Several interictal features were highly correlated, including spikes and relative entropy (see Supplementary Fig. 2 and Supplementary Results). We performed a secondary analysis in which we excluded 15 unilateral patients who did not undergo surgery or who had one-year Engel outcomes > 1. We observed similar trends in this smaller patient cohort (Supplementary Fig. 3).

### fMRI BOLD connectivity AI also distinguishes SOZ lateralities

**Figure 3:**
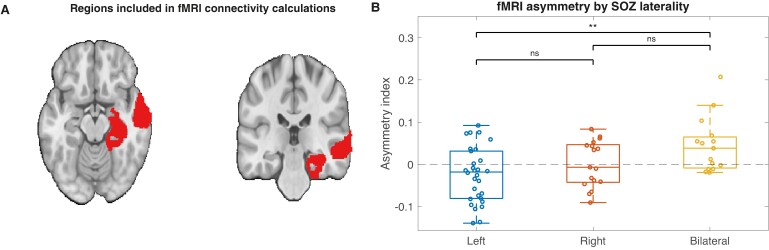
**Comparison of fMRI asymmetry index (AI) across seizure onset zone (SOZ) lateralities.** (**A**) Regions (Brainnetome parcels) included in fMRI connectivity calculations (shown for the left temporal lobe only). (**B**) fMRI AI by SOZ laterality. fMRI AI distinguished left from bilateral SOZs more easily than right from bilateral SOZs. There was a significant difference in fMRI connectivity AI between temporal lobe epilepsy lateralities (*n* = 62 patients, ANOVA: *F*(2,59) = 6.1, *P* = 0.004, *η*^2^ = 0.17, Fig. 2D). Only the difference between the left and bilateral SOZ group was significant after correcting for multiple comparisons (*P* = 0.002).

We next asked if differences in interictal connectivity between TLE lateralities existed across modalities beyond just EEG. We tested whether fMRI connectivity asymmetry similarly distinguished left, right and bilateral SOZs. Figure 3A shows the left temporal Brainnetome atlas regions included for fMRI connectivity analysis. There was a significant difference in fMRI connectivity AI between TLE lateralities (ANOVA: *F*(2,59) = 6.1, *P* = 0.004, *η*^2^ = 0.17, Fig. 3B). Only the difference between the left and bilateral SOZ group was significant after correcting for multiple comparisons (*P* = 0.002). Similar to the result for interictal EEG data, this suggests that fMRI temporal lobe connectivity AI distinguishes patients with left from bilateral SOZs, but cannot distinguish between patients with right and bilateral SOZs.

### A classifier incorporating interictal EEG features predicts SOZ laterality

**Figure 4:**
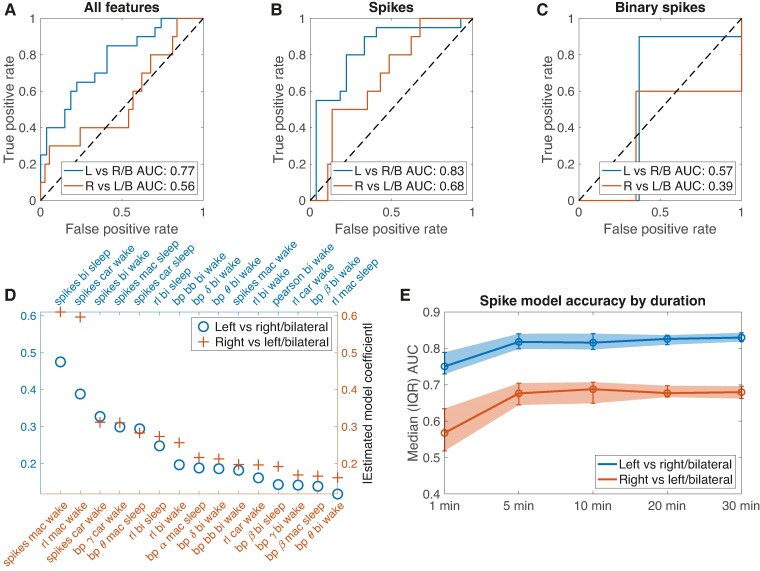
**Classifier to distinguish SOZ lateralities using interictal IEEG asymmetry.** (**A**) Internal cross-validation performance for models trained on all interictal IEEG features (*n* = 47 patients). (**B**) Internal cross-validation performance for models trained on only spike rate asymmetry index (common average reference) (*n* = 47 patients). (**C**) Internal cross-validation performance for models trained on only spike rate asymmetry index (common average reference), binarized such that 1 = more spikes on the left and 0 = more spikes on the right (*n* = 47 patients). (**D**) Absolute value of the estimated model coefficients for the full feature set models. mac, machine reference; car, common average reference; bi, bipolar reference; spikes, spike rate; re, relative entropy; bp, bandpower; rl, spike recruitment latency (a measure of spike timing). Greek letters denote canonical frequency bands. (**E**) Model areas under the curve (AUCs) for models trained on subsampled durations of spike rate data.

We tested whether interictal features predict SOZ laterality in unseen patients. The areas under the curve (AUCs) of the receiver operating characteristic curve of the left- and right-sided internal cross-validation models trained on all features were 0.77 and 0.56, respectively (Fig. 4A). A model trained on only spike rates (restricted to CAR reference and sleep in order to have a single feature) achieved higher AUCs [0.83 and 0.68 for the left and right models, respectively (Fig. 4B)]. Finally, a model trained only on binary spike rates indicating whether there were more spikes on the left or the right performed poorly [AUC of 0.57 and 0.39, respectively (Fig. 4C)]. Spike rates, spike timing and bandpower were the most important features for both the left- and the right-sided models (Fig. 4D).

**Figure 5:**
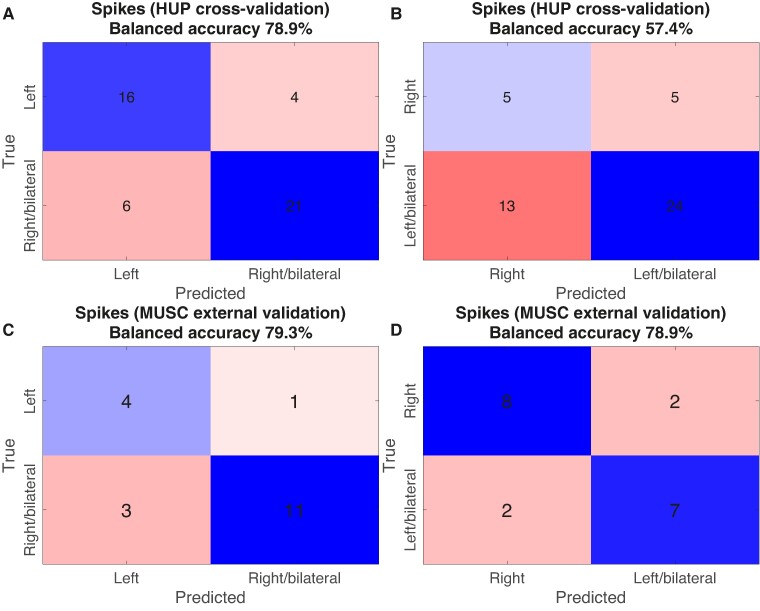
**Confusion matrix showing accuracies for spike-rate only models to predict SOZ lateralities.** (**A** and **B**) Confusion matrices (internal cross-validation) for spike-rate only models (from Fig. 4B) at the optimal model operating point for the models predicting left versus right/bilateral SOZ and right versus left/bilateral SOZ, respectively. (**C** and **D**) Confusion matrices for the spike-rate only models (from Fig. 4B) for the models predicting left versus right/bilateral SOZ and right versus left/bilateral SOZ, respectively, applied to the external testing set.

We further probed the accuracy of the spike-rate only model. Confusion matrices for the left- and right-sided models at the optimal operating points are shown in Fig. 5A and B. The balanced accuracy was 78.9% for the model predicting left versus right/bilateral SOZ, and 57.4% for the model predicting right versus left/bilateral SOZ. Model accuracies rise quickly with duration sampled, achieving an accuracy similar to the full-duration models with 5 minutes of sampling (Fig. 4E). Finally, we tested how the spike-only models performed in the external MUSC dataset. The balanced accuracies were 79.3% and 78.9% for the left-sided and right-sided models, respectively (Fig. 5C and D).

These results suggest that models using only spike rate asymmetry accurately distinguished left from right or bilateral SOZs in both internal cross-validation and in a separate institution's test dataset. However, although right-sided SOZs could be distinguished from left/bilateral SOZs in the external dataset set, they were not well-classified in the internal validation dataset. Results were similar, although with higher AUCs across all models, when we restricted analysis of unilateral HUP patients to be those with Engel 1 surgical outcomes to build and internally validate the SOZ laterality classifier (Supplementary Figs 4 and 5). Results were also similar when we used spikes detected in bipolar and machine references to build the SOZ laterality classifier (Supplementary Figs 6–9). There was no significant association between model accuracy and mesial temporal (*n* = 42) versus temporal neocortical (*n* = 9) localization for either the left- or right-sided model [left-sided model odds-ratio: 0.5 (95% CI 0.1–2.6), *P* = 0.42; right-sided model: 0.7 (0.2–3.3), *P* = 0.69]. Similarly, there was no significant difference in model accuracy between patients with temporal lobe lesions on MRI (*n* = 27) and patients whose MRI had other lesions or no lesion (*n* = 39) [left-sided model odds-ratio: 0.3 (95% CI 0.1–1.3), *P* = 0.13; right-sided model: 1.8 (0.6–4.9), *P* = 0.30].

### Concordance between spike-predicted laterality and surgical laterality is higher for patients with good surgical outcomes

**Figure 6:**
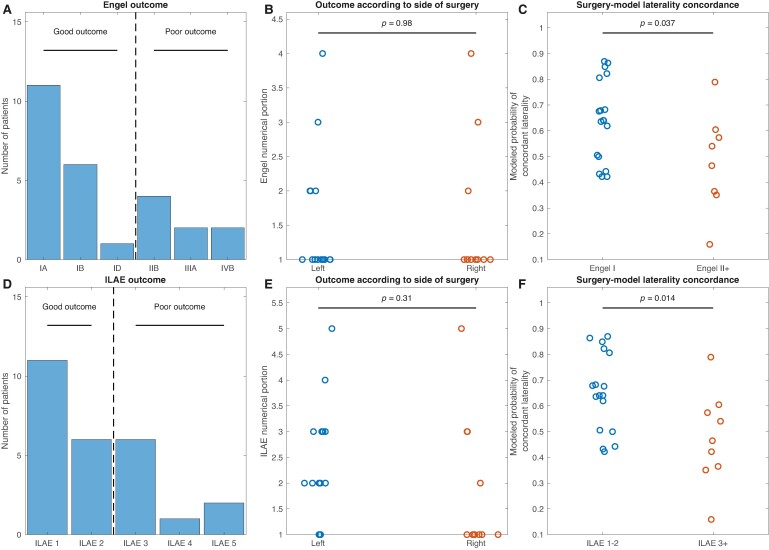
**Surgical outcome prediction.** (**A** and **D**) Distribution of one-year surgical outcomes using the Engel and ILAE (International League Against Epilepsy) classification schemes, respectively (*n* = 26 patients who underwent surgery and had one-year outcome data). (**B** and **E**) The numerical portion of the Engel and ILAE surgical outcome score, respectively, according to the side of surgery. There was no significant difference in outcomes between left- and right-sided surgeries. Engel: *t*(24) = −0.0, *P* = 0.98; ILAE: *t*(24) = 1.0, *P* = 0.31. (**C** and **F**) The modelled probability of SOZ laterality concordant with the side of surgery for patients who had a good (Engel 1 or ILAE 1–2) or poor (Engel II+ or ILAE 3+) surgical outcome. Patients who had a good surgical outcome had higher laterality-concordant model probabilities than patients who had a poor surgical outcome (Engel: *t*(24) = 2.2, *P* = 0.037, ILAE: *t*(24) = 2.6, *P* = 0.014).

Eighteen of 26 (69.2%) patients had good one-year Engel outcomes (Engel I), and 8 of 26 (30.8%) had poor Engel outcomes (Engel 2+) (Fig. 6A). Seventeen of 26 (65.4%) patients had good one-year ILAE outcomes (ILAE 1–2), and 9 of 26 (34.6%) had poor ILAE outcomes (ILAE 3+) (Fig. 6D). The means of the numerical portions of the outcome scales were similar for patients who underwent left- versus right-sided surgeries (Fig. 6B and E; Engel: *t*(24) = −0.0, *P* = 0.98; ILAE: *t*(24) = 1.0, *P* = 0.31). Left-sided surgeries were disproportionately ablations (12 ablations versus 3 resections), and right-sided surgeries were more often resections (4 ablations versus 7 resections). We hypothesized that patients with a good surgical outcome would have a higher modelled probability of SOZ laterality concordant with the side of surgery. We identified the spike rate model corresponding to the side of surgery. Mean concordant model probability was significantly higher in patients with good Engel outcomes (mean (SD) 0.64 (0.16)) than in patients with poor Engel outcomes (0.48 (0.19)) (*t*(24) = 2.2, *P* = 0.037) (Fig. 6C), and in patients with good ILAE outcomes (0.65 (0.15)) than in patients with poor ILAE outcomes (0.47 (0.18)) (*t*(24) = 2.6, *P* = 0.014) (Fig. 6F). Finally, we measured how well modelled TLE laterality predictions predicted surgical outcome in unseen patients. We trained a logistic regression model to predict good or bad surgical outcome using the modelled probability of SOZ laterality concordant with the side of surgery as the model input. The performance of the model validated using leave-one-out classification had an AUC of 0.65 for predicting Engel outcome and 0.71 for predicting ILAE outcome (Supplementary Fig. 10). Together, these results suggest that a model trained to predict the SOZ using spike rate asymmetry is also associated with surgical outcome, although it has only modest ability to predict outcome. Results were similar when we used spikes detected in bipolar and machine references (Supplementary Figs 11 and 12).

## Discussion

We compared a large array of previously published interictal intracranial EEG features across TLE patients with different SOZ lateralities. Left-sided TLE was easier to distinguish than right-sided TLE, a finding we also observed in a separate interictal modality of fMRI connectivity. A model based on just spike rates predicts SOZ laterality in unseen patients.

### Left-sided SOZs are easier to identify than right-sided SOZs

Our univariate analysis of interictal EEG features (Fig. 2B), our machine learning algorithm (Fig. 4A–C) and our fMRI analysis (Fig. 3B) all found that left-sided SOZs were easier to distinguish than right-sided SOZs in our internal HUP cohort. It is possible that this finding is specific to patients who undergo intracranial EEG recording: patients with well-localized right-sided TLE are more likely to immediately pursue a temporal lobectomy given lower concern about cognitive impact from resecting the non-dominant hemisphere; thus, patients with right-sided TLE who undergo intracranial EEG implantation may have challenging features to their localization. This hypothesis may also explain the discrepancy in right-sided model performance across HUP and MUSC if these two centres have different approaches to select patients with suspected TLE for intracranial EEG monitoring. This hypothesis was not supported by our finding that patients with left-sided and right-sided TLE had similar surgical outcomes, however, this is confounded by the fact that patients with left TLE more often underwent laser ablation, which is known to have lower rates of seizure freedom than temporal lobectomy.^37,38^ An alternate hypothesis is that right TLE may be associated with broader network dysfunction. This hypothesis is conceivable due to differences in language networks,^39,40^ and is supported by functional and structural neuroimaging studies demonstrating more widespread abnormalities in right-sided TLE.^41-45^

### A multivariate set of interictal features is worse than spikes alone

A model using only spike rates distinguished SOZ lateralities with higher accuracy than a model testing many interictal features that have been previously studied in intracranial EEG literature (Fig. 4A and B). We suspect that the better performance of the spike-only model arises from the fact that spikes are the most important feature (Fig. 4D), and the benefit of adding more features is outweighed by the downside of overfitting to this more complex feature set. We included intracranial EEG features that have been reported to localize seizure generators in prior studies.^11-16^ Relative entropy, a recently-described metric that compares the distribution of amplitudes between EEG signals on electrode pairs,^13,16^ was the non-spike feature that differed the most across lateralities (Fig. 2A), although it was also strongly correlated with spike rates (Supplementary Fig. 1A and C). Overall, we found no clear lateralizing value in these quantitative features beyond spikes alone. In univariate analyses, features in sleep had the highest effect size at lateralizing the SOZ (Fig. 2A). In our multivariate model, features from both sleep and wake had the highest feature importance (Fig. 4D). Prior studies found that models trained on spikes in sleep outperformed those trained on spikes in wakeful states.^13,35^

### Spike rate asymmetry identifies left-sided TLE in both internal and external cohorts

The spike-only model accurately identified left-sided TLE in both the internal cross-validation and external cohorts (Fig. 5A and C), suggesting good external validity. The performance of the model trained to identify right-sided TLE was low in the internal cross-validation cohort but high in the external cohort (Fig. 5B and D). Part of the discrepancy in performance between the two cohorts may result from the class imbalance between the two sites: HUP had more patients with left-sided TLE and MUSC had more patients with right-sided TLE. The lower number of right-sided patients in the HUP cohort may have hurt performance of this model, although we attempted to mitigate this by weighting the misclassification cost by the inverse of the number of patients. Alternatively, it may be related to different approaches to surgical planning in the two centres, as discussed above.

Importantly, clinician designations of SOZ laterality may be incorrect: a patient may have only unilateral seizures during the intracranial evaluation and receive a diagnosis of unilateral TLE, but actually have bilateral TLE.^4^ We found that the concordance between the spike model-predicted SOZ laterality and the side of surgery was associated with good one-year surgical outcomes (Fig. 6). This result suggests that a model incorporating spike rates reveals information about the laterality of the true seizure generators.

Prior studies found that spike rates in scalp EEG lateralize TLE, and in particular that when spikes are mostly unilateral, the side of the spikes usually agrees with the side of the clinician-defined epilepsy.^46-50^ Our study adds to these findings by (i) incorporating interictal intracranial EEG data; (ii) providing a quantitative framework to interpret non-unilateral spike rates; and (iii) evaluating patients undergoing intracranial EEG, who likely have more challenging lateralization than patients with TLE more generally. A future direction is to understand if quantitative analysis of interictal scalp EEG data is as effective as invasive data in these patients, which may help avoid intracranial evaluation in some cases.

### Limitations

Both our internal validation cohort and our external validation cohort have relatively few patients, which limits the external validity. We had only six overlapping patients between our fMRI dataset and our IEEG dataset, which precluded us from assessing the complementary information they could provide in a multimodal approach. Also, because our machine learning algorithm does not predict SOZ localization, we cannot identify patients whose seizure generators were correctly lateralized, but incorrectly localized. This is a particular limitation for those patients who underwent laser ablation, where precisely localizing seizure generators is crucial. We were underpowered to compare the ability to predict surgical outcomes according to type of surgery. Given the heterogeneity in the cohort of patients who underwent resection or ablation, as well as the expected difference in seizure freedom according to type of surgery, this is an important limitation in our outcome analysis. Although we demonstrated relative temporal stability in performance of the models throughout the Epilepsy Monitoring Unit admission, we cannot exclude longer-term fluctuations that occur over weeks to months.^51,52^ The timing of intracranial monitoring in relation to these longer-term cycles may affect the ability of interictal features to localize seizure generators.

Our choices of connectivity measures, frequency ranges and filter settings were guided by those from relevant prior publications. However, a more exhaustive parameter search may reveal important activity excluded from our analysis that may distinguish epileptic from non-epileptic networks. We used only a single automated algorithm to detect spikes, and this algorithm was originally validated in a cohort of children with neocortical epilepsy.^26^ Although we separately validated the positive predictive value in our cohort, we did not test sensitivity, as this would have required a prohibitively exhaustive validation to account for the long duration sampled and the change in spike rates and location over time.^23^ Other detectors with higher reported accuracy may yield improved overall model performance.^53^ Also, although we aim to study interictal features, it is possible that subtle subclinical seizures that went unrecognized by the clinical team would have been coded as interictal data. Also, we do not exclude immediate preictal or postictal periods, and these time periods may be different from true interictal periods. Regarding sleep staging, the SleepSEEG algorithm we used to automatically stage sleep was validated in a patient population with heterogeneous depth electrode recordings,^20^ and we performed additional validation in a subsequent study in our group.^35^ However, it is unknown how the implant strategy or epilepsy localization affects algorithm performance. Therefore it is possible that sleep staging is less reliable in the TLE population. We were unable to perform further validation in this study because most patients did not have scalp EEG to permit manual sleep staging. Furthermore, we made no attempt to remove sleep-specific activity such as K complexes or sleep spindles from our analysis. Therefore, it is possible that normal sleep architecture could be mistaken for interictal spikes. Others have found altered sleep architecture in epilepsy, which may be captured in quantitative features such as bandpower, and thus could be used to help lateralize TLE.^54^

### Clinical translation

As spike rates can be counted manually or with commercially available software, only short durations are required, and the spike rate model accurately predicted left-sided TLE in both our internal and external cohorts, we believe that the spike rate model is clinically useful. The accuracy at predicting right-sided TLE was low in the internal cohort, and so we would not recommend using this model to predict right-sided TLE without further validation in larger external cohorts. One potential clinical application of our left-sided model would be to support or oppose a hypothesis of unilateral left TLE when deciding whether to proceed with resection/ablation versus to implant a responsive neurostimulator device with the goal of capturing chronic data to confirm laterality.^4^ In the setting of a moderate pre-test probability of left TLE, finding a low probability of left TLE in this model may prompt clinicians to pursue device placement. Given the particular risk to cognition with left temporal resection and ablation,^55-57^ having ancillary data to guide surgical planning is critical in patients with suspected left TLE.

To promote reproducibility of our results, we provide a free online calculator to apply our model. Clinicians and researchers can input spike rates from non-rapid eye movement sleep in the left and right temporal lobes, and the calculator returns the spike rate AI, along with the predicted probabilities of left-sided and right-sided SOZs derived from the left- and right-sided models. Our goal is for clinicians to be able to use this model, along with other electroclinical data, to help guide surgical planning.

## Conclusion

The results of this study support the hypothesis that interictal abnormalities lateralize to the side of seizure generators in TLE. Furthermore, a simple model using spike rates can accurately predict left-sided SOZs in unseen patients. Given the limitations of using seizures to lateralize seizure generators, we believe that our study provides additional motivation to use spike rates to aid in surgical planning in patients with suspected TLE.

## Supplementary Material

fcae284_Supplementary_Data

## Data Availability

Raw EEG data are available on ieeg.org. All code used to perform analyses, along with an intermediate dataset containing electrode contact-level features, is publicly available on https://github.com/penn-cnt/cnt_tle_laterality/. The online calculator to predict SOZ laterality given temporal lobe spike rates is available on https://penn-cnt.github.io/epilepsy_lateralization/.
